# Investigation on mechanical properties of nickel open cell metal foam after heat treatment

**DOI:** 10.1038/s41598-023-42857-w

**Published:** 2023-10-03

**Authors:** Y. Shajari, L. Nikzad, M. Razavi

**Affiliations:** Department of Ceramic, Materials and Energy Research Center, Karaj, Iran

**Keywords:** Engineering, Materials science

## Abstract

This investigation aims to assess the mechanical behavior and energy absorption properties of the open-cell nickel foams. The metal foams produced by electroforming of nickel on PU foams, also a heat treatment has applied to evaporate the PU foam, then a uniaxial compression test was applied to measure maximum compressive strength, energy absorption density, efficiency, and normalized stresses. The results indicate that compared with typical open-cell nickel foams and polymer precursors when the electroforming time is 12 h and a heat treatment has applied, the aforementioned properties of the metal foams had a significant improvement. Improvement of properties will change by increasing the time of electroforming. The heat treatment improved the energy absorption density of open-cell nickel foams for 3.7 times. For the best sample which is a metal foam with 12 h of electroforming with heat treatment the first maximum compressive strength, energy absorption density, and energy absorption efficiency reach 1.84 (MPa), 3.29 (mJ/mm^3^), and 73%, respectively.

## Introduction

The idea of metal foams refers back to natural porous medias such as wood, bone and other living tissues. These novel materials find their special role in today’s different industries and they are still developing by broad range of experiments. The metallic cellular materials could divide to two categories, open-cell and closed-cell metal foams. Closed-cell metal foams has found their industrial applications among, functionally graded materials, foam filled tubes, acoustic dampers, gas silencers and military armors. The closed-cell type of metallic porous materials has shown great specific energy absorptions, energy absorption density, and marvelous dynamic responses to impacts with strengthened structures which has a lot of great studies and are a well-known engineering material^1–9^.

Among all kinds of porous medias, open cell metal foams with high porosity are of importance. Ever since the advent of open cell metal foams prominent properties have been widely investigated and reported in different studies. Three-dimensional open cell structures and considerable specific surface areas of Open-cell foams, results in unique features of permeability, high tortuosity, high damping properties, large surface-to-volume ratio, excellent electrically and thermally conductivity; thus, they can be used for thermal management in electronics, battery electrodes, catalyst carriers, exhaust gas recirculation (EGR) filters and lightweight structures^10–16^. Open-cell nickel foams are widely used in electrodes for battery applications. They can be utilized as containers for electrolytes and collectors of electric current. In general, the foams can be subjected to mechanical loading conditions, therefore, high strength is considered as a crucial factor and investigating of the mechanical behavior of metal foams is considered imperative^12,17–20^. The mechanical behavior of cellular structures is governed by their internal architecture^21^. Various new methods and manufacturing technologies have developed recently for the production of these recyclable materials which depends on the characteristics expected from the porous media. Feasible processes for manufacturing metal foams reviewed by Banhart^22^ which mainly included infiltration casting, gas entrapment, electro-deposition methods, and powder compact melting^23–28^.

Characterization of mechanical or physical properties of Cellular metals can be done in different tests^29^. There are two main methods to examine cellular material: from an atomistic or molecular viewpoint a cellular material is a construction consisting of a multitude of struts, membranes or other elements which themselves have the mechanical properties of some bulk metal. In general, one can distinguish non-destructive and destructive methods according to whether the foam is experiencing a permanent deformation or remains unchanged or only minimally affected during characterization. These methods consist of density measurements, penetration measurements, X-ray radiography and radioscopy, X-ray computed tomography, Eddy-current sensoring, Acoustic measurements, Vibrational analysis, porosimetry and permeametry, Electrical and thermal conductivity measurements, Optical image analysis, mechanical testing and Corrosion testing.

The compressive properties of the nickel foams including yield strength, elastic modulus, energy absorption density and energy absorption efficiency were calculated by Fan^30^ and the results show that the compressive properties of yield strength, elastic modulus and energy absorption density increase with the increase of relative density of nickel foams. Feshat et al.^31^ used polyurethane foam as a precursor and electrodeposition method as the production method to investigate the mechanical behavior of nickel foams through simulation in order to be utilized in lithium battery applications. Mohammad Shaheta8 reported tensile strength of nickel foams of about (0.65 MPa). Furthermore, Levy et al.^32^ produced an open-cell nickel foam and examined the effect of carbon coatings on the properties of these foams, which are widely used in supercapacitors.

The lack of morphological tools able to characterize the real microstructures of open cell metal foams limits the knowledge of pertinent geometrical parameters able to visualize the structure of the foams^33^. X-ray tomography has recently proved to be an applicable tool allowing the characterization of the microstructure of the foam materials. To illustrate large deformations of cellular solids such as important buckling, bending or fracture events in a non-destructive way X-ray tomography is known to be powerful^34^.

Open cell nickel foams were produced using electrodeposition process and the aim of this investigation was recognizing the effect of heat treatment on the macrostructure, microstructure, and mechanical properties of nickel foams with different strut thicknesses.

## Materials and methods

In this examination, the precursor of polyurethane 10 pore per inch (PPI) foam was used for manufacturing open cell nickel foams. PU foams were cut in the 2 × 3 × 3 cm and immersed in an alkaline solution containing sodium hydroxide, sodium carbonate, and three sodium phosphate. Then the precursor was placed in a solution of potassium manganate and sulfuric acid, washed with distilled water and immersed in the ${\text{SnCl}}_{2}$ + HCl solution. In the chemical deposition process, Ag was plated on the surface of the foams. In the electrochemical deposition process Ag-electrolessed foams were nickel plated at (1.50 v DC) voltage with a current of (1.7 A) for 4, 6, 8, 10, 12 h. This process led to a nickel layer with different thickness which is the main variable in this investigation. From now on samples without heat treatment with different times of electrodeposition are named Ni1-4, 6, 8, 10, 12 h and samples with heat treatment are named Ni2–4, 6, 8, 10, 12 h. The weight and apparent density of nickel foams are mentioned in Table 1.

**Table 1:** Physical properties of foams.

Samples	Weight (g)	Apparent density (${\text{g}}/{\text{cm}}^{3}$)
Ni1-4 h	4.21	0.23
Ni1-6 h	4.73	0.26
Ni1-8 h	7.21	0.40
Ni1-10 h	9.92	0.55
Ni1-12 h	10.35	0.57

In order to investigate the effects of heat treatment on mechanical properties and microstructure of electrodeposited nickel foam; heat treatment of nickel foams was done in Muffle Furnaces in 410 $^\circ{\rm C}$ for 1 h in Ar 99.99% atmosphere.

According to ISO 13314 standard^35^ for flat porous materials, a compression test was designed and performed with Instron machine with a 5 ton capacity to evaluate the compressive behavior of open cell nickel foams. The uniaxial compression test was done with the strain rate of 0.013 s^−1^ at room temperature. SEM images were taken by Field Emission Electron Microscope (FE-SEM), which has a resolution of (1.5 nm) at (15 kV) and (4.5 nm) at (1 kV).

## Results and discussion

### Microstructural evaluation

**Figure 1:**
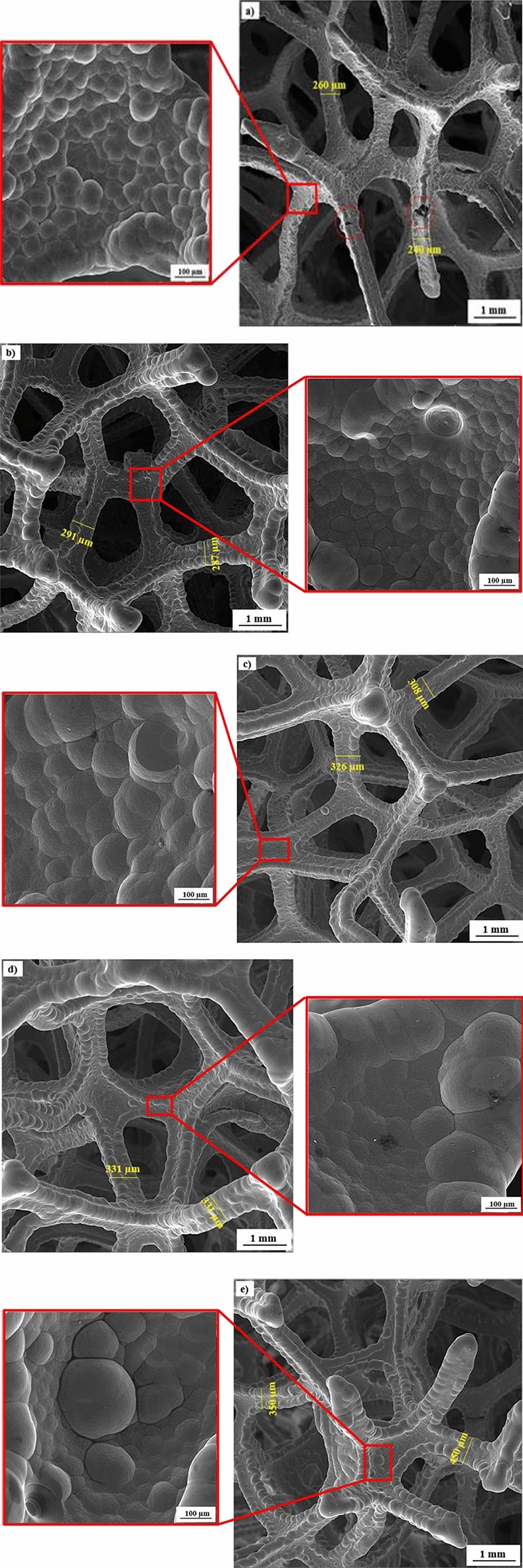
SEM images of (**a**) Ni1-4 h, (**b**) Ni1-6 h, (**c**) Ni1-6 h, (**d**) Ni1-10 h, (**e**) Ni-12 h.

In this part, the microstructure and morphology of the open-cell metal foams have been investigated. As can be seen in the Fig. 1, which illustrates Ni1-4 h, Ni1-6 h and Ni1-8 h, Ni1-10 h, Ni-12 h the coated nickel microstructures are arranged in the form of cauliflower and a relatively homogenous level has been achieved.

This homogeneity can be seen in the uniformity of the size of congresses and their orderly arrangement. In all samples, the conglomerates are larger at nodes, this is due to the flow leakage at the edges and the increase in the deposition rate in those areas due to the occurrence of the dog bone phenomenon. The Fig. 1a corresponds to a sample that has been placed in a nickel-plating solution for 4 h, parts of the surface can be seen that nickel has not covered the entire surface of the polyurethane foam as well as other parts, which can be one of the reasons for the lower strength of this sample, because after the evaporation of the carbon layer, this layer practically has the ability to collapse and will have a very destructive effect on the mechanical properties. This effect is destructive even when the internal carbon layer is not removed, because it acts like a crack in static and dynamic loading and leads to premature failure. With the increase of the coating time, the thickness of the nickel layer increased, which means an increase in the diameter of the struts. It is clear that by increasing the time of nickel electrodeposition, the areas that were weak in coating have been completely fixed, which is clearly visible in the structure that has been electrolyzed for 10 and 12 h which can be seen in Fig. 1d,e. Also, the sizes of struts and holes have been measured in the images, because the size of the struts is one of the most important parameters that has a direct effect on all properties of foams such as mechanical, acoustic, electrical and thermal properties.

It is clear that with the increase of electrolysis time, the thickness of the layer increases and the rate of this increase from 4 to 12 h is 58%. This increase in thickness is initially due to the high germination rate, but later the growth phenomenon prevails over the germination. This is the reason that in the morphology of the congress-sized cauliflower has increased.

In the structure of foams produced by electrodeposition method, the deposition layer is formed in semi-amorphous or crystalline form. By performing heat treatment along with the removal of the polymer precursor, the structure is completely crystallized due to crystallization, and then with recrystallization, the crystals are arranged again and regularly, which increases the mechanical performance, toughness, and shock absorption. The reason is the increase of the boundary between the grains. As a result of new seed germination and its growth during heat treatment, superficial cauliflowers may increase in size. As a result of this growth in congresses due to the increase in the number of seeds and their growth, a strain is created in the network, which can lead to the hardening of the network and increase the toughness.

### Mechanical characterization

**Figure 2:**
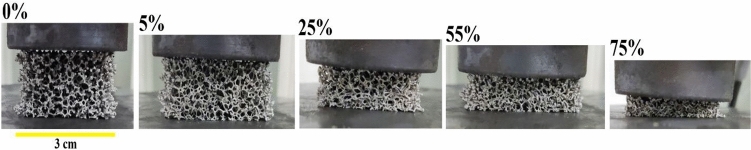
Ni1-12 h during compression test in terms of strain percentages.

In the current study, the compressive behavior, the energy absorption properties, and the normalized stresses of metal foams have been investigated. The compressive behavior of Ni foams was presented in Fig. 2. According to Fig. 2, the compression of these porous materials has no visible fracture and the porous structure shows a uniform compressive behavior up to the densification point and last steps of compression test.

**Figure 3:**
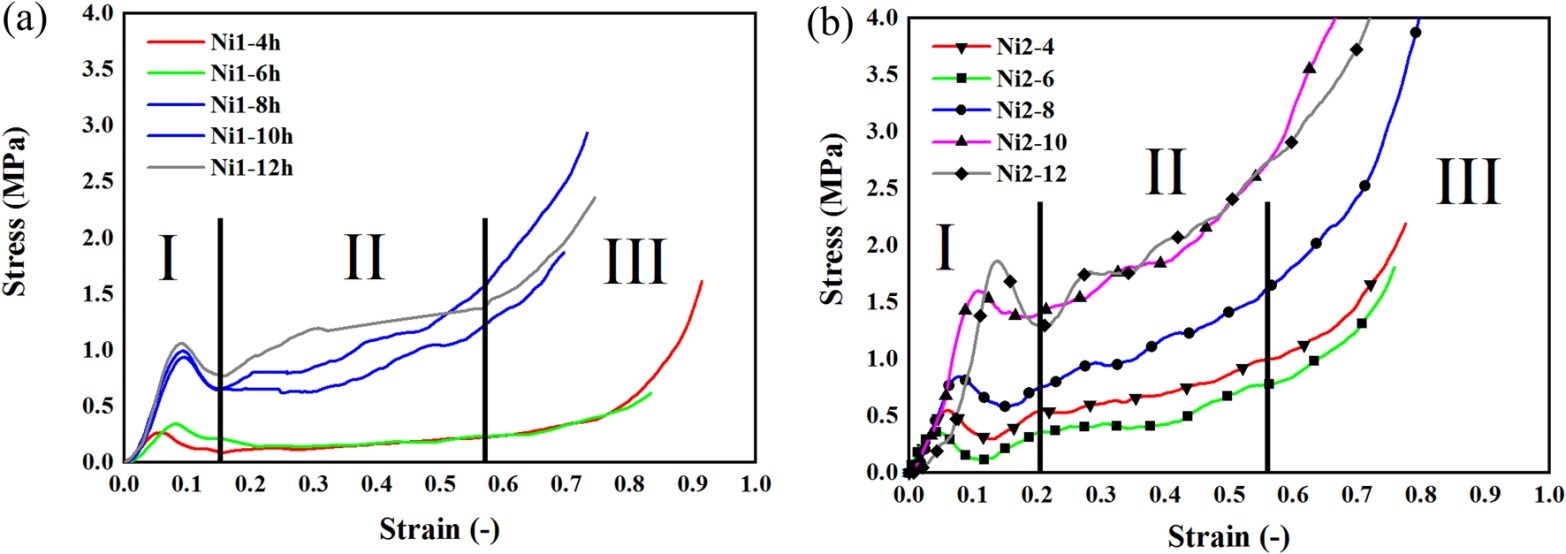
Stress strain behavior of the nickel foams for (**a**) samples without heat treatment (Ni1), (**b**) samples with heat treatment (Ni2).

According to Fig. 3, the time of electroforming has affected the mechanical properties of the Nickel foams in a good way. The Ni1 samples could be grouped in two group, the first group shows a maximum reaction force around 1 kN, and the other group shows a maximum tolerated force less than 0.5 kN. On the other hand, Ni2 samples show a more predictable behavior with a maximum tolerated force around 2 kN.

The force displacement curves transformed to stress strain curves and are presented in Fig. 3. The Fig. 3 illustrates the compressive behavior of the nickel foams (Ni1 and Ni2) of the current study, First region is the first tolerated stresses which contain a first massive rise in stress values, which is called the first maximum compressive strength 12 (yield strength).This part is the start of deformation. First ruptures happen after the highest value of stress in this part of the diagram. Second region, plateau zone, which it contains node fractures of the metallic foams. Lastly, the diagram reaches the densification of the metal foams, which the behavior of the metal porous material is going to reach the behavior of the bulk material. According to Fig. 3b, the heat treatment affects the compressive behavior of metal foams and improved the yield point of the metal foams and the plateau region of the diagram which could lead to a better energy absorption and compressive behavior for the current materials.

**Figure 4:**
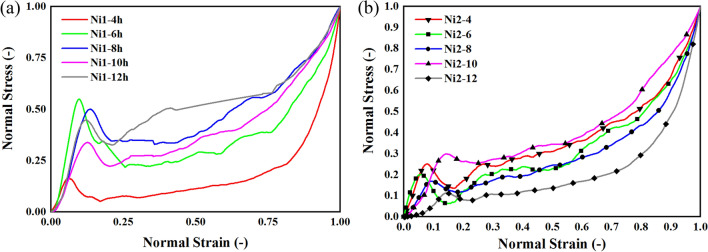
Normalized stresses for (**a**) samples without heat treatment (Ni1), and (**b**) samples with heat treatment (Ni2).

Investigating the normalized stresses are a new approach that is used to better understand the mechanical response and characteristics of porous materials^36^. Equation (1), normalized the tolerated stresses to reach a better and comparative factor. this normalized parameter is presented in Fig. 4.

1 $$NS=\sum\limits _{i=1}^{n}\frac{{\sigma }_{{t}_{i}}}{\max\left[0,{\sigma }_{{t}_{i}}\right]}.$$

According to Fig. 4, both groups, samples with heat-treatment and samples without heat-treatment, has experienced the yield point before strains of 0.25. Nevertheless, heat treated samples has shown a more uniform compressive behavior which resulted in better energy absorptions and also, they have a better plateau region. Moreover, samples with lower electroforming time shows a better mechanical response after receiving heat treatments. Furthermore, the effect of electroforming time is pretty visible in the cycle of improvement for all samples with or without heat treatments.

The energetic properties studied for the present samples are the absorbed energy, complementary energy and energy absorption efficiency. The absorbed energy can be calculated through Eq. (2) and the complementary energy could be calculated through Eq. (3). The energy absorption density or strain energy is the area under the force displacement diagram^9^.

2 $$u=\frac{U}{V}=\int \limits_{0}^{x}\frac{F.dx}{V}=\int \limits_{0}^{x}\frac{F.dx}{A{.X}_{0}}=\int \limits_{0}^{\varepsilon }\sigma .d\varepsilon ,$$

3 $${u}^{*}=\frac{{U}^{*}}{V}=\int \limits_{0}^{F}\frac{x.dF}{V}=\int \limits_{0}^{F}\frac{x.dF}{{X}_{0}.A}=\int \limits_{0}^{\sigma }\varepsilon .d\sigma ,$$

where $u$ is the energy absorption density, u* is complementary energy and $V$ is the volume of foams.

**Figure 5:**
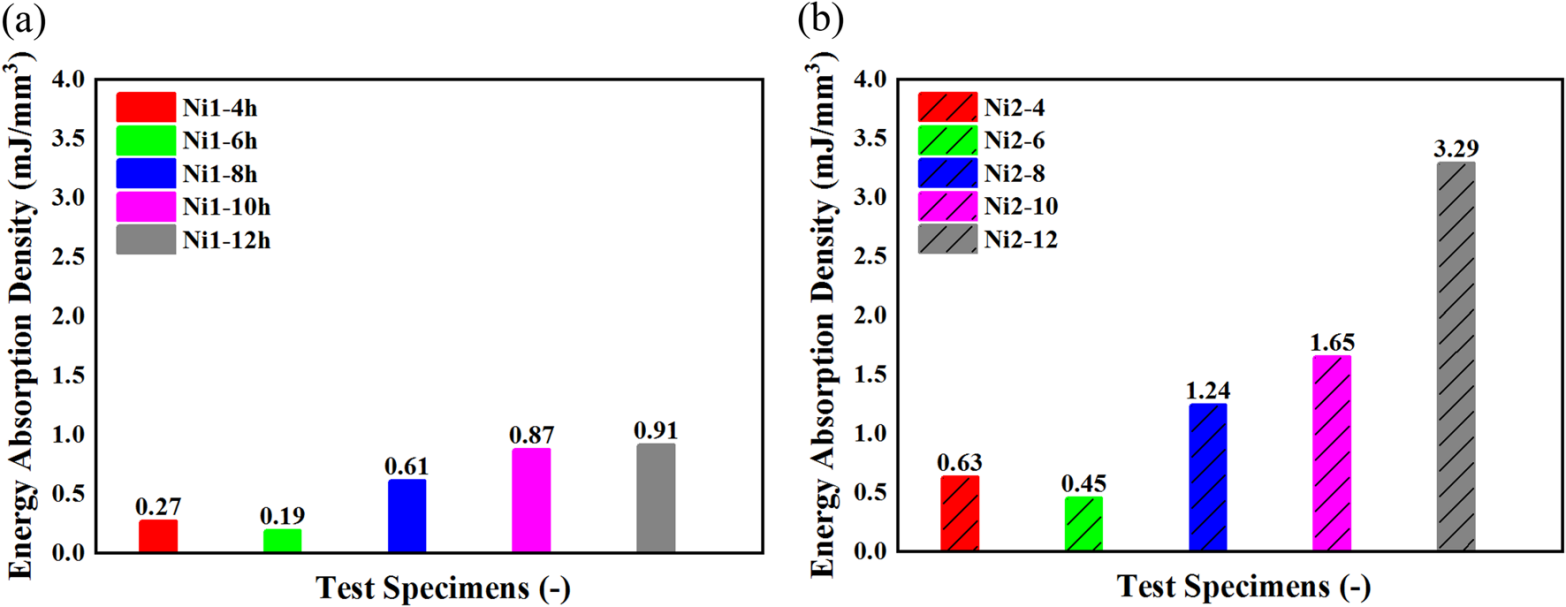
Energy absorption density for (**a**) samples without heat treatment, and (**b**) samples with heat treatments.

The energy absorption density is calculated through Eq. (2) and presented in Fig. 5. The improvement of the amount of energy absorption is a function of electroforming time for these metallic foams except the samples with 6 h of electroforming. However, the sample with 12 h of electroforming and a heat treatment has tripled the amount of energy absorption density for samples with 12 h of electroforming with no heat treatments. This improvement could lead to a new type of metal foams which has a well capacity of energy absorption.

**Figure 6:**
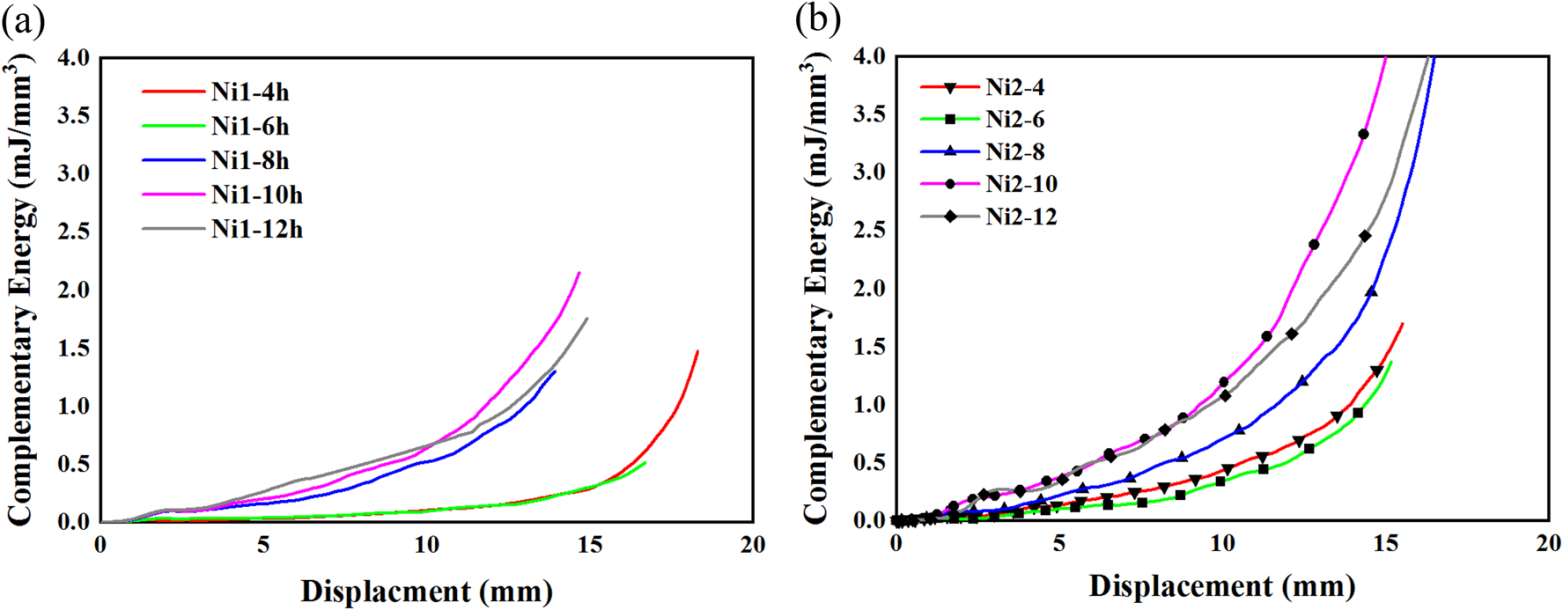
Energy absorption density for (**a**) samples without heat treatment, and (**b**) samples with heat treatments.

The complementary energy is calculated through Eq. (3) and presented in Fig. 6. Similar to other mechanical properties of these materials the complementary energy is improved by applying the heat treatment to Ni1 samples. The complementary strain is the potential of a material to absorb energy, in this case, the heat treatment improved the potential of these materials in an ascending order and caused a huge improvement for these materials.

**Figure 7:**
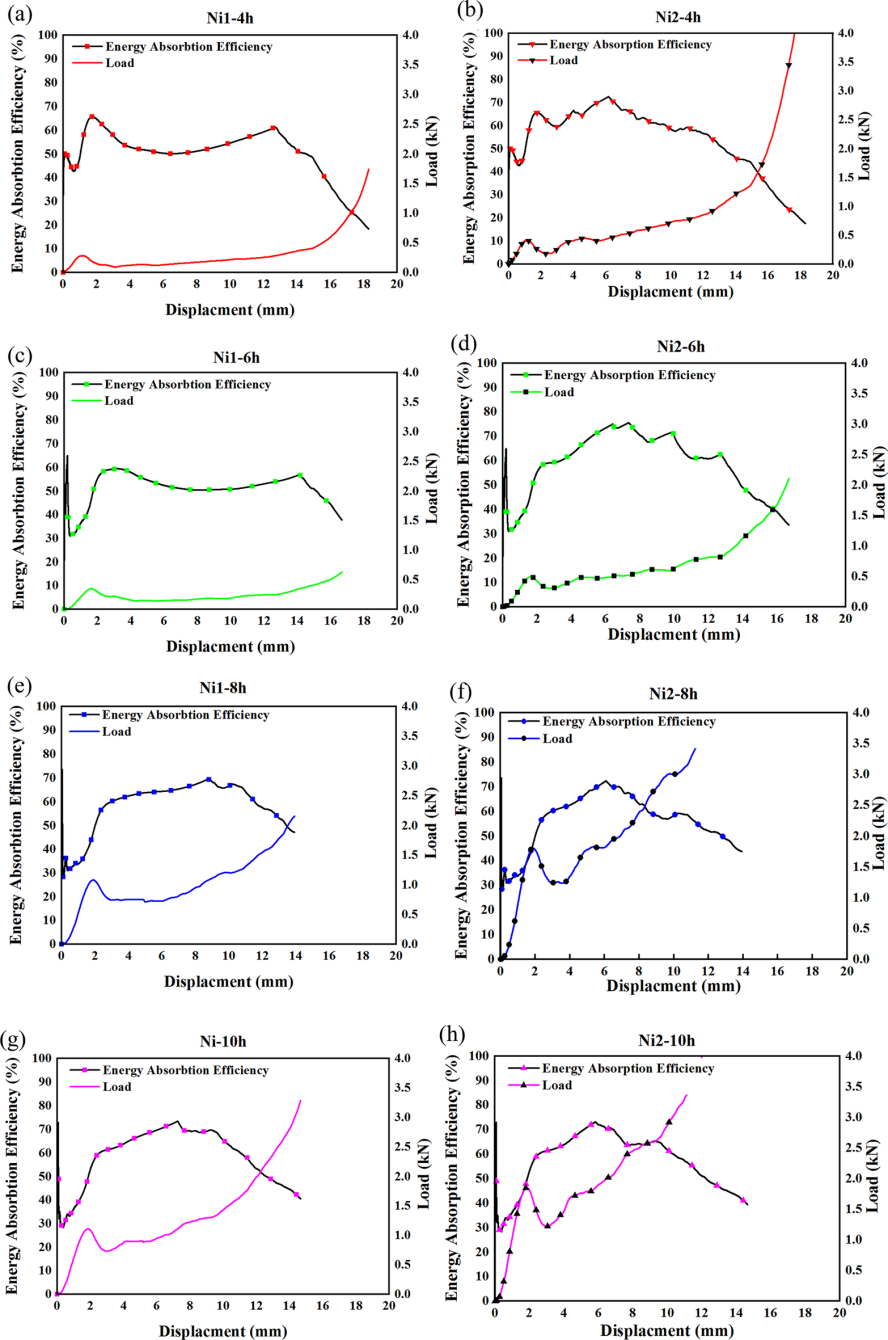

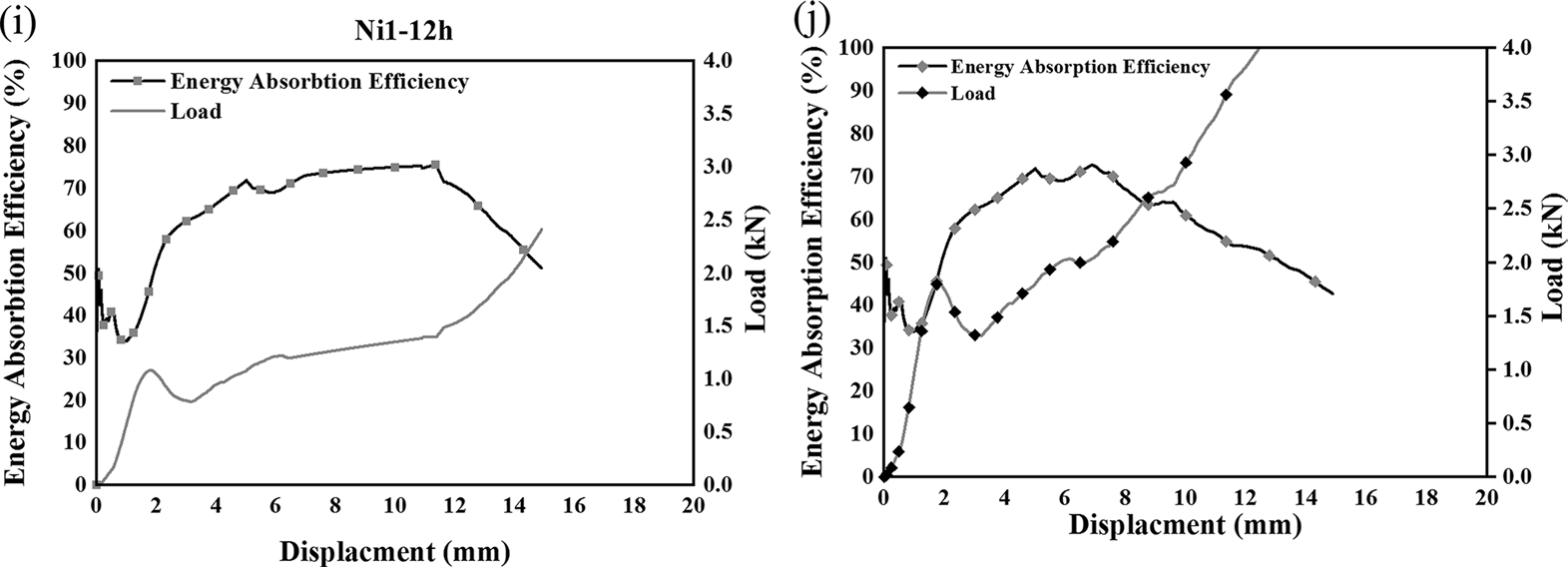
Energy absorption efficiency of Ni1 (**a,c,e,g,i**) and Ni2 (**b,d,f,h,j**).

η expresses the rate of energy absorbed at a particular strain relative to the total amount of absorbed energy and calculated through Eq. (4), and plotted concerning displacement in Fig. 7.

4 $$\eta =\frac{1}{{\sigma }_{m}{\varepsilon }_{m}}\int \limits_{0}^{{\varepsilon }_{m}}\sigma d\varepsilon ,$$

where $\eta$ is Energy absorption efficiency when strain is ${\varepsilon }_{m}$ and ${\sigma }_{m}$ is stress at a certain strain ${\varepsilon }_{m}$.

According to Fig. 7, which contains the energy absorption efficiency for all Ni1 and Ni2 samples, approximately the same behavior is observed for both samples at the same electroforming time. The energy absorption efficiency curve reaches a peak, then a fall in efficiency is followed by a stable region in the curve. Its concentration is around the yield point of samples. However, the heat treatment improved the energy absorption efficiency of the samples, because they have experienced more strains compared to the samples with no heat treatments. This behavior could lead to a more flexible metal foam which could be very beneficial in energy absorption or high strain applications.

## Conclusion

The open-cell metal foam of current study manufactured with electroforming and had heat-treatments. This metal foams have the following characterizations.

1. The samples have electroformed for 4, 6, 8, 10, and 12 h. It is clear that with the increase of electrolysis time, the thickness of the layer increases and the rate of this increase from 4 to 12 h is 58%. Moreover, a heat treatment process has applied to Ni2 samples. This heat treatment resulted in improving the homogeneity of grains and it actually refined the grain boundaries in a good way.
2. The yield point of the open-cell nickel foams has affected by the heat treatment. Ni1-12 h is the best sample amount the samples without heat treatments and Ni2-12 h is the best sample amount the heat-treated samples. The yield point of Ni1-12 h is 1.03 MPa and the yield point of Ni2-12 h is 1.76 MPa. The increase in yield point of samples is the effect of the heat treatment on microstructure of Ni1 samples.
3. All open-cell metal foams of the current study show a similar mechanical behavior in terms of normalized stresses. All samples have a yield point around the strain of 0.25 in terms of normalized stress–strain diagrams.
4. The heat treatment improved the mechanical properties of nickel metal foams especially in terms of energy absorption density. The energy absorption density of the open-cell nickel foams improved from 0.91 to 3.45 mJ mm^−3^ which is about 3.7 times.

## Data Availability

The datasets generated during and/or analyzed during the current study are available from the corresponding author on reasonable request.
